# The effect of implementing undergraduate competency-based medical education on students’ knowledge acquisition, clinical performance and perceived preparedness for practice: a comparative study

**DOI:** 10.1186/1472-6920-13-76

**Published:** 2013-05-27

**Authors:** Wouter Kerdijk, Jos W Snoek, Elisabeth A van Hell, Janke Cohen-Schotanus

**Affiliations:** 1 Center for Research and Innovation in Medical Education, University of Groningen and University Medical Center Groningen, Ant. Deusinglaan 1, FC40, 9713 AV, Groningen, The Netherlands; 2 Institute for Medical Education, University of Groningen and University Medical Center Groningen, Groningen, The Netherlands

**Keywords:** Medical education, Competency-based education, Undergraduate medical education, Competence, Curriculum development, Curriculum comparison, Active learning, Clinical performance, Self-efficacy, Progress test

## Abstract

**Background:**

Little is known about the gains and losses associated with the implementation of undergraduate competency-based medical education. Therefore, we compared knowledge acquisition, clinical performance and perceived preparedness for practice of students from a competency-based active learning (CBAL) curriculum and a prior active learning (AL) curriculum.

**Methods:**

We included two cohorts of both the AL curriculum (n = 453) and the CBAL curriculum (n = 372). Knowledge acquisition was determined by benchmarking each cohort on 24 interuniversity progress tests against parallel cohorts of two other medical schools. Differences in knowledge acquisition were determined comparing the number of times CBAL and AL cohorts scored significantly higher or lower on progress tests. Clinical performance was operationalized as students’ mean clerkship grade. Perceived preparedness for practice was assessed using a survey.

**Results:**

The CBAL cohorts demonstrated relatively lower knowledge acquisition than the AL cohorts during the first study years, but not at the end of their studies. We found no significant differences in clinical performance. Concerning perceived preparedness for practice we found no significant differences except that students from the CBAL curriculum felt better prepared for ‘putting a patient problem in a broad context of political, sociological, cultural and economic factors’ than students from the AL curriculum.

**Conclusions:**

Our data do not support the assumption that competency-based education results in graduates who are better prepared for medical practice. More research is needed before we can draw generalizable conclusions on the potential of undergraduate competency-based medical education.

## Background

In response to societal concerns about the role of doctors in contemporary healthcare, competency-based medical education is receiving increasing attention worldwide [1-9]. Its underlying assumption is that competency-based medical education results in doctors who are better prepared for medical practice [10]. In Canada and the United States, the national accreditation councils have implemented competency-based criteria for *postgraduate* medical education [1,11]. Additionally, a competency framework has been proposed and guidelines have been developed for *undergraduate* competency-based medical education [5,12,13]. In the European Union, as part of the Bologna process, all medical schools are required to base their undergraduate curricula on a clear and well-defined set of competencies [14]. A major focus of competency-based curricula is to facilitate students’ development of competencies, demonstrable abilities consisting of knowledge, skills and professional behaviour. Consequently, when implementing competency-based medical education, curriculum time has to be reserved for students’ competency development [2,15]. This means there will be less time available for existing activities of preceding curricula. Therefore, such a reallocation of time may not only result in the facilitation of competency development but may also impair students’ development in other areas. To our knowledge, the gains and losses associated with implementing undergraduate competency-based curricula are still unknown. Therefore, we examined undergraduate medical students’ knowledge acquisition, clinical performance and perceived preparedness for medical practice for two curricula – a competency-based active learning (CBAL) curriculum and its predecessor, a regular active learning (AL) curriculum.

Undergraduate medical curricula usually have a set duration. When implementing competency-based education, curriculum time has to be reserved so students can develop their competencies. The time reserved for activities aimed at competency development will usually come at the expense of time previously reserved for knowledge acquisition [15]. This reallocation of time may negatively affect students’ knowledge acquisition in a competency-based curriculum. Although medical students’ knowledge has not been found to be an immediate predictor of clinical performance, it does impact clinical performance indirectly [16]. To allow for well-informed decisions about curriculum innovations, it should be clear whether implementing undergraduate competency-based medical education leads to knowledge loss among medical students.

One of the key forces behind competency-based medical education is the public call for medical curricula to reflect the needs of contemporary medical practice [1,15,17,18]. Therefore, competency frameworks comprehensively reflect what a competent doctor should be able to demonstrate in practice, [19] and should benefit students’ preparation for medical practice. Throughout competency-based curricula, relevant competencies and their relation with practice are continuously emphasized which helps students to understand what is expected of them during medical training and in medical practice [3,12]. Consequently, students should feel better prepared for practice which, in turn, is a prerequisite for self-efficacy – the extent to which a person believes that he or she can successfully fulfil a specific task in a specific context. Self-efficacy is of key importance for developing competence and autonomy in practice [20,21]. We expected students from a CBAL curriculum to feel better prepared for medical practice and to perform better during clerkships than students from an AL curriculum, where the presence of an underlying competency framework is less explicit.

The possible gains and losses associated with the implementation of an undergraduate competency-based medical curriculum provide valuable information for future curriculum development and add to the theory of competency-based medical education. Therefore, we examined the influence of implementing a competency-based curriculum on medical students’ knowledge acquisition, clinical performance and perceived preparedness for medical practice.

## Methods

### Context

The AL and the CBAL curriculum were developed and implemented at the University of Groningen, The Netherlands. Characteristics of both curricula are presented in Table 1. The CBAL curriculum was implemented in September 2003 and focuses on seven areas of competence: communication, clinical problem-solving, using basic knowledge and science, patient investigation, patient management, social and community contexts of health care and reflection [22].

**Table 1:** Characteristics of the Active Learning and Competency-Based Active Learning curriculum at the UMCG

	**AL curriculum**	**CBAL curriculum**
**Active learning**	Emphasis on active learning in small groups	Emphasis on active learning in small groups
**Focus on competencies**	0% curriculum time allocated specifically for competency development	15% curriculum time allocated specifically for competency development
	No portfolio or small group sessions aimed at competency development	Portfolio and small group sessions aimed at competency development
	Purpose of a course is communicated	Purpose of a course and the related competencies are communicated
**Clerkships**	80 weeks of clinical experience	80 weeks of clinical experience
	Rotational duration is 1–8 weeks	Rotational duration 4–5 weeks
	22 rotations	15 rotations
	Last clerkship rotation entails an elective of 13 weeks	Last clerkship rotation entails an elective of 20 weeks

In both curricula, active learning principles are applied to facilitate *knowledge acquisition*. Students learn in small groups, collaborate with their peers and engage in self-directed learning. Teachers and tutors fulfil a coaching and facilitating role [23].

Learning methods and the amount of time reserved for *skills training* are similar in both curricula. However, in the AL curriculum skills training is divided over smaller courses throughout the preclinical phase, whereas skills training in the CBAL curriculum is concentrated in the first year of the clinical phase. During this year, five-week periods of skills training in the clinical training centre are alternated with five-week clerkship rotations. The purpose of this alternation is to ease the transition from the preclinical to the clinical phase by helping students develop their skills, just in time, to apply them in practice and to further integrate them with knowledge and professional behaviour [24].

The main difference between the two curricula lies in the emphasis on *competency development*. In the CBAL curriculum, the link between the purpose of each course and relevant competencies are clearly communicated throughout the course. This is not the case in the AL curriculum. Furthermore, 15% of the total CBAL curriculum time is reserved specifically for small group sessions aimed at competency development. Time for these sessions is created by diminishing the number of small group sessions originally aimed at knowledge acquisition in the AL curriculum. The total curriculum time remains the same.

Throughout the preclinical phase of the CBAL curriculum, small group sessions for competency development are based on students’ experiences in practice and assignments related to each area of competence. An example of such an assignment is that first-year students, unfamiliar with medical practice, have to describe the qualities of a good doctor. In their third study year the students have to repeat this assignment, and reflect on what they have learnt and experienced in the meantime. Other assignments are related to activities in intramural or extramural practice – for example an internship in a nursing home or consecutive interviews with a chronically ill patient. The competency development sessions are facilitated by a senior faculty member and are scheduled six to eight times a year. Additionally, students have to collect their assignments in a portfolio, on which they receive feedback bi-annually.

During the clinical phase, sessions aimed at competency development are scheduled 24 times a year. During these sessions students discuss their own experiences and certain themes in relation to their development (for example cultural diversity or dealing with death). In addition to assignments related to these meetings, students have to keep track of a personal development plan in their portfolio in which they formulate learning goals based on the areas of competence. During the clinical phase the portfolio is evaluated twice a year in an interview with a senior staff member.

The curriculum time reserved for clerkships is 80 weeks in both curricula. Students in the CBAL curriculum rotate through fewer disciplines than students in the AL curriculum. In the AL curriculum, clerkship duration varies between one and eight weeks and students rotate through 22 disciplines. When designing the CBAL curriculum we felt that the aim of clerkships shifted from experiencing as many disciplines as possible towards a balance between diversity and the stability of surroundings to support students’ competency development. Consequently, in the CBAL curriculum, the minimum duration for clerkship rotations was extended to 4 weeks to allow sufficient time for students to work on their competencies. Consequently, the number of clerkship rotations was reduced to 15. Furthermore, the last clerkship rotation entailed a clinical elective of which the duration was increased from 13 weeks in the AL curriculum to 20 weeks in the CBAL curriculum.

### Participants

Undergraduate medical education in The Netherlands lasts 6 years. We included students who graduated within 7 years from the start of the last 2 cohorts of the AL curriculum (2001/2002 and 2002/2003; N = 453) and the first 2 cohorts of the CBAL curriculum (2003/2004 and 2004/2005; N = 372).

### Ethical statement

Data were gathered during the time that, under Dutch law, educational studies were exempt from Institutional Board Review. At that time, no ethical review board for medical educational research existed in the Netherlands. However, data gathering was carried out in accordance with established ethical standards and the Declaration of Helsinki [25-27]. The privacy policy of the University of Groningen states that student records can be used for research purposes, as long as reports cannot be traced back to individual students [28]. In accordance with this privacy policy, anonimyzed data were derived from the university administration.

### Instruments

Knowledge acquisition was assessed by benchmarking our cohorts’ scores on the Dutch interuniversity progress test (IPT) against those of parallel cohorts from two other Dutch medical schools with similar cohort sizes (approximately 250 students per cohort). All cohorts sat the IPT four times per year at the same time, i.e. 24 tests per cohort. The IPT is based on the Dutch National Blueprint for the Medical Curriculum, and is designed to asses “the end objectives of undergraduate medical training as far as knowledge is concerned” [29,30]. Each progress test contains 200 multiple choice questions and is constructed to reflect the entire domain of medical knowledge. The IPT is not related to the curriculum of one particular institution [30]. The reason for benchmarking against two other medical schools was that all students sat exactly the same tests at the same point in their education. IPT benchmarking is especially suitable for analysing effects of curriculum changes because, at the time of our study, admittance to medical schools in the Netherlands was still primarily determined by a national lottery system [31]. This system guarantees an intake of first-year students which is very similar across medical schools with regard to past performance, age, gender and motivation to study medicine [32]. Over the period of our study the medical schools used for comparison had not changed their curricula.

Clinical performance was operationalized as students’ average clerkship grade. In both curricula clinical assessment was identical: each clerkship grade was based on several mini-CEX scores. Mini-CEX scores are sufficiently reliable to estimate clinical competence [33]. In both curricula, grades were given on a 10-point scale.

To measure perceived preparedness for medical practice, we used data from an internal quality control survey, measuring how prepared students feel in each area of competence. Perceived preparedness was measured for 33 competencies (Table 2), using a 5-point scale (1 = ‘insufficiently prepared’, 5 = ‘excellently prepared’). Our medical school considers a mean score between 4 and 5 as excellently prepared, between 3 and 4 as well-prepared and below 3 as insufficiently prepared.

**Table 2:** Means, standard deviations and t-statistics for perceived preparedness of graduates from two curricula

	**Active learning curriculum**		**Competency-based active learning curriculum**		
	***N *= 172**		***N *= 177**		
	**Mean**	**(SD)**	**Mean**	**(SD)**	***t*-test**
**Communication** (α = 0.80)	4.29	(0.44)	4.33	(0.41)	–.787
Communicating with a patient	4.48	(0.58)	4.55	(0.57)	–1.112
Treating a patient with respect and confidentiality	4.51	(0.56)	4.62	(0.5)	–1.842
Working together with colleagues	4.36	(0.59)	4.32	(0.54)	.637
Accepting the expertise of others	4.31	(0.56)	4.36	(0.54)	–.717
Building and maintaining a doctor-patient relationship	4.00	(0.74)	3.98	(0.76)	.210
Efficiently consulting with colleagues and other health care professionals	4.08	(0.63)	4.14	(0.55)	–.872
**Clinical problem-solving** (α = 0.70)	3.98	(0.48)	3.90	(0.4)	1.594
Using a systematic approach to a patient problem	4.15	(0.6)	4.09	(0.54)	.904
Interpreting problem descriptions, patient history, physical examinations and other findings	3.99	(0.57)	3.88	(0.54)	1.804
Making a differential diagnosis	3.88	(0.61)	3.77	(0.57)	1.641
Deciding which information about treatment should be provided to the patient	3.90	(0.75)	3.87	(0.66)	.405
**Using basic knowledge and science** (α = 0.86)	3.53	(0.67)	3.62	(0.62)	–1.340
Conducting scientific research	3.45	(0.86)	3.63	(0.77)	–2.015
Approaching scientific information critically	3.58	(0.85)	3.68	(0.71)	–1.184
Converting scientific information into effective policy	3.52	(0.75)	3.53	(0.73)	–.133
Justifying conduct based on a scientific argumentation	3.56	(0.74)	3.65	(0.74)	–1.083
**Patient investigation** (α = 0.70)	3.91	(0.48)	3.86	(0.4)	1.114
Diagnosing a patient problem	3.91	(0.57)	3.82	(0.56)	1.553
Documenting relevant information	3.99	(0.7)	4.02	(0.59)	–.330
Performing a physical examination	4.02	(0.63)	3.99	(0.58)	.355
performing of medical skills expected from an MD	3.73	(0.64)	3.61	(0.6)	1.786
**Patient management** (α = 0.84)	3.77	(0.55)	3.68	(0.52)	1.555
Determining a founded and suitable treatment	3.69	(0.66)	3.59	(0.61)	1.454
Executing a treatment plan	3.66	(0.7)	3.51	(0.72)	1.946
Monitoring the effects of a treatment plan	3.58	(0.71)	3.48	(0.78)	1.197
Adjusting a treatment plan	3.56	(0.74)	3.39	(0.71)	2.284
Hold an effective and respectful consultation with a patient	4.35	(0.61)	4.42	(0.56)	–1.168
**Social and community contexts of health care** (α = 0.75)	3.66	(0.49)	3.77	(0.48)	–2.193
Placing a patient problem in a broad context of political, sociological, cultural and economic factors	3.75	(0.77)	3.97	(0.66)	−2.899*
Being aware of the consequences of the patient problem for the patients environment	4.03	(0.64)	4.11	(0.57)	–1.210
Having knowledge of factors that influence health and disease at societal level	3.56	(0.68)	3.72	(0.66)	–2.281
Promoting health of patient and society as a whole	3.61	(0.71)	3.69	(0.71)	–.968
Following relevant legal regulations	3.33	(0.77)	3.37	(0.75)	–.486
**Reflection** (α = 0.73)	3.77	(0.51)	3.85	(0.47)	−1.423
Recognizing and acknowledging one’s own shortcomings	4.15	(0.56)	4.26	(0.55)	−1.903
Combining work life with private life	3.61	(0.84)	3.58	(0.84)	.357
Dealing with ethical dilemmas	3.78	(0.67)	3.82	(0.63)	–.529
Formulating and carrying out a personal education plan	3.62	(0.79)	3.79	(0.77)	–1.980
Reflecting on the conduct of colleagues	3.72	(0.67)	3.78	(0.62)	–.931

* = Significant at the α = .01 level.

### Analysis

To analyse students’ knowledge acquisition, we used a method based on the first steps in the longitudinal benchmarking methods described by Muijtjens et al. [34]. We compared our students’ average score to those of the students from the other medical schools, using t-tests. The 24 means were plotted in a graph for each cohort. When our students scored significantly higher or lower, a ↑ or ↓ was drawn in the graph, respectively. A Bonferroni correction was used to compensate for the high number of tests and effect sizes were calculated.

We compared clinical performance and perceived preparedness for medical practice in the CBAL and AL curriculum using independent sample t-tests. With regard to perceived preparedness for medical practice, we first calculated the internal consistency of the scales using Cronbach’s α. Subsequently, curricula were compared on the mean scores for both items and scales using an α of 0.01 and effect sizes were calculated.

## Results

### Knowledge acquisition

**Figure 1:**
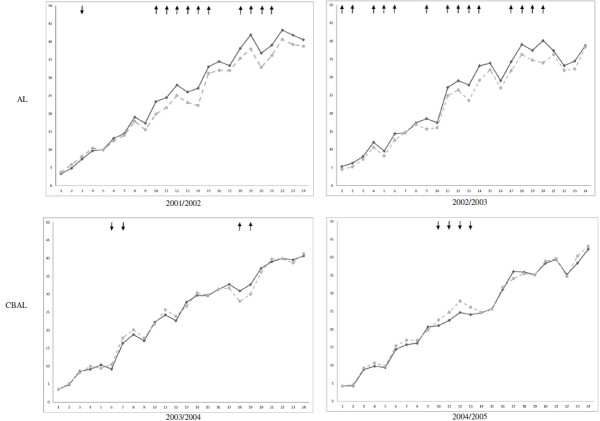
**Mean progress test scores of UMCG cohorts compared to those from two other medical schools.** Mean scores (Y-axis) of the UMCG (solid line) cohorts from the AL curriculum (2001/2002 and 2002/2003) and the CBAL curriculum (2003/2004 and 2004/2005) compared to the combined mean scores of the cohorts from two other medical schools (dashed line) on 24 progress tests (X-axis). A downwards arrow (↓) or an upwards arrow (↑) marks the UMCG scoring significantly lower or higher than the other two schools, respectively.

The AL cohorts scored significantly higher on 10 (2001–2002; ES 0.30–0.57) and 14 progress tests (2002–2003; ES 0.27–0.66) and significantly lower on 1 progress test (2001–2002; ES 0.31) than cohorts from the other two medical schools. The CBAL cohorts scored significantly higher on 2 progress tests (2003–2004; ES 0.30 and 0.34) and significantly lower on 2 (2003–2004; ES 0.24 and 0.27) and 4 progress tests (2004–2005; ES 0.23–0.44) than cohorts from the other two medical schools (Figure 1). None of the 4 cohorts scored significantly different on the last three tests of the final year.

### Clinical performance

We did not find a significant difference between the clinical performance of students from the CBAL curriculum (Mean = 7.91; SD = 0.28) and the AL curriculum (Mean = 7.87; SD = 0.35; t(823) = −1.540; p = 0.124).

### Perceived preparedness for medical practice

Of the CBAL and AL curriculum, 177 (48%) and 172 students (46%) completed the survey, respectively. Respondents and non-respondents were similar in gender distribution (74% and 70% female respondents, respectively) and mean clinical performance (Mean = 7.89; SD = 0.29 and Mean = 7.88; SD = 0.33, respectively). The internal consistency of the scales ranged from 0.70 to 0.86 (Table 2). Graduates from the CBAL curriculum felt excellently prepared for 10 and well prepared for 23 competencies. Graduates from the AL curriculum felt excellently prepared for 11 and well prepared for 22 competencies. Students from both curricula felt best prepared to treat a patient with respect and confidentiality (Mean_AL_ = 4.51; Mean_CBAL_ = 4.62) and felt worst prepared for following relevant legal regulations (Mean_AL_ = 3.33; Mean_CBAL_ = 3.37). At scale level, students felt excellently prepared for communication and well prepared in the other areas of competence. We found no significant differences at scale level. At item level, students from the CBAL curriculum felt better prepared for putting a patient problem in a broad context of political, sociological, cultural and economic factors (t(347) = −2.90; p = 0.004; ES = 0.31).

## Discussion

The aim of our study was to analyse the effects of the implementation of a competency-based active learning curriculum (CBAL) as compared to the previous active learning curriculum (AL). Using progress test results, we found relatively less knowledge acquisition in the first years of the CBAL curriculum than in the first years of the AL curriculum. However, we did not find such difference in the final year. Graduates who had been trained in a CBAL curriculum did not score higher on clinical performance nor did they feel better prepared for medical practice.

Implementing competency-based education requires that curriculum time is reserved for activities that facilitate competency development. As more time is allocated to the development of competencies, less time will be devoted to other curricular activities. In undergraduate curricula these activities usually involve knowledge acquisition. As a consequence, implementing a CBAL curriculum bears the risk of knowledge loss. We analysed students’ knowledge acquisition by comparing the scores of CBAL and AL cohorts on 48 progress tests to those of parallel cohorts from two other medical schools, which had not changed their curriculum during the time of our study. Our assumption was that if our students’ relative position remained unchanged, there would have been no knowledge loss. In comparison to the cohorts of the other medical schools, our AL cohorts scored significantly higher on 50% of the progress tests (24 out of 48), whereas our CBAL cohorts scored significantly higher on only 4% of the tests (2 out of 48). However, at the end of undergraduate education the CBAL and the AL cohorts demonstrated similar knowledge acquisition. The effect sizes of the differences were small to medium. As we interpret the outcomes concerning the progress tests as trends per cohort rather than results per test, we feel the effect sizes are large enough to conclude that students in the AL curriculum show higher knowledge acquisition than the students in the CBAL curriculum in the first years of their undergraduate education. Reserving time for competency development at the expense of time reserved for knowledge acquisition, seems to lead to lower knowledge acquisition in the short term, but not in the long term.

Throughout the medical curriculum, knowledge plays an important part in expertise development [16,35,36]. As the CBAL cohorts seldom scored lower than the comparison cohorts and no long-term differences were found, we consider a permanent negative impact of implementing competency-based education on student learning and expertise development unlikely. An explanation for this finding might be that the clinical environment encourages students to regulate their own learning [37]. During clerkships students are repeatedly stimulated to remedy deficiencies in medical knowledge. Undergraduate students’ prior knowledge deficiencies appear to be overcome during their clerkships.

We expected CBAL students to perform better in clinical practice than AL students. However, we did not find a significant difference, which may indicate that implementation of competency-based education has no effect on clinical performance. A possible explanation for this finding may be that all students must be competent to work with real patients at the start of their clerkships, which restricts differentiation among students [38]. This homogeneity among clerks may explain why our clerks were mainly scored at the high end of the scale by their supervisors. Thus, we may have found no difference between the CBAL and the AL curriculum due to a restriction of range, caused by the requirements for entering the clinical phase.

We expected the CBAL students to feel better prepared for medical practice. To analyze students’ perceived preparedness we used survey data collected at graduation. The only difference we found between the two curricula is related to one of the core aims of competency-based medical education. Students from the CBAL curriculum felt better prepared to put a patient problem in a broad context of political, sociological, cultural and economic factors, which is in line with the aim to educate medical professionals who are sufficiently responsive to societal needs [1,15,17,18]. It is also in line with the focus of competency-based medical education on the development of professionals in a societal context [2,3,5,12,19]. However, we were unable to demonstrate any other effects of the implementation of competency-based education on students’ perceived preparedness.

The fact that we did not find a general increase in student’s perceived preparedness for medical practice may be related to the educational tools we implemented to facilitate competency development: portfolio use and explicit communication of competencies and their underlying framework. A recent study by Sargeant et al. revealed that explicit communication of competencies and the use of portfolios help students to achieve informed self-assessment [39]. Students in the CBAL curriculum are frequently informed of what is expected of them and they are explicitly stimulated to reflect on their performance, to remedy their deficiencies and to formulate points of improvement. The awareness that follows from these activities may help students to become increasingly conscious of their deficiencies. Possibly, CBAL students were more aware of their competencies and incompetencies than AL students, which is an important step in the development of competence [40]. Consequently, the CBAL students may have underestimated their preparedness for practice as compared to AL students. Further research is needed to analyse the influence of implementing a CBAL curriculum on students’ reflectiveness and, subsequently, on their self-assessment.

A possible limitation of our study is that it is a single-site study, which affects the generalizability of our results. However, comparing curricula from the same institution has the advantage that most variables can be controlled. When the CBAL curriculum was introduced, teaching staff and learning methods remained largely unchanged. Consequently, our data have been gathered in the same context which increases the likelihood that possible effects can be attributed to the implementation of the CBAL curriculum. However, more studies are needed before generalizable conclusions can be drawn. Furthermore, our measurement of perceived preparedness had a limited response of 47%. However, the respondents and non-respondents were similar in gender distribution and clinical performance, which suggests that the sample was representative of the overall population.

Another limitation of our study might be that the measures we used – knowledge acquisition, clinical performance and perceived preparedness – are not specific to competency-based education. One could argue that for studying the effectiveness of competency-based education, measures are needed that fit conceptually. In our curricula, clinical competence was mainly assessed using global judgements. For research purposes, specific judgements may do more justice to the complexity of competencies. However, in this study such information was not available.

Finally, our study was limited to measurements during the course of undergraduate medical training and at graduation. Possibly, effects of competency-based education will become more apparent after graduation, in actual practice. Further research is needed to determine the long-term effects of implementing competency-based education at the undergraduate level. Despite the limitations of our study, we consider our outcome measures relevant because of their relation to performance in actual medical practice [16,21]. Irrespective of the curriculum, medical graduates are expected to have sufficient knowledge and skills to practice professionally. Therefore, our study yields valuable information on the effect of implementing undergraduate competency-based education.

## Conclusion

Implementing competency-based education in our undergraduate medical curriculum neither resulted in clerks who scored higher on clinical performance nor in graduates who felt better prepared for practice at the end of their training. Our study shows that there is some knowledge loss in the first study years of a CBAL curriculum as compared to the previous curriculum. Our study does not support the assumption that competency-based curricula result in graduates who are better prepared for medical practice. However, since this is one of the first studies in the field, it is too early to draw generalizable conclusions. More research is needed before we can conclude whether or not competency-based education meets the high expectations associated with its widespread implementation.

## Competing interests

The authors declare that they have no competing interests.

## Authors’ contributions

All authors were involved in the conception and design of this study. WK gathered and analysed the data. All authors interpreted the data together and were involved in drafting and revising the manuscript. All approved the final manuscript.

## Authors’ information

Wouter Kerdijk, Msc, is a Psychologist and Researcher in Medical Education at the Center for Research and Innovation in Medical Education, University of Groningen and University Medical Center Groningen, Groningen, The Netherlands.

Jos W. Snoek, MD, PhD, neurologist, is professor in Clinical Education and Director of the Master of Medical Science Program at the Institute for Medical Education, University of Groningen and University Medical Center Groningen, Groningen, The Netherlands.

Elisabeth A. Van Hell, PhD, is an Educationalist at the Institute for Medical Education, University of Groningen and University Medical Center Groningen, Groningen, The Netherlands.

Janke Cohen-Schotanus, PhD, is professor in Research in Medical Education and Head of the Center for Research and Innovation in Medical Education, University of Groningen and University Medical Center Groningen, Groningen, The Netherlands.

## Pre-publication history

The pre-publication history for this paper can be accessed here:

http://www.biomedcentral.com/1472-6920/13/76/prepub
